# Exploring Current Health Policies on Rare Diseases in Greece: A Narrative Review

**DOI:** 10.1155/ijpe/5572290

**Published:** 2025-10-21

**Authors:** Pelagia Tsitsani, Theodora Papamitsou, Maria Malliarou, Elpidoforos S. Soteriades

**Affiliations:** ^1^Department of Paediatrics, General Hospital of Katerini, Katerini, Central Macedonia, Greece; ^2^Department of Healthcare Management, School of Economics and Management, Open University of Cyprus, Nicosia, Nicosia District, Cyprus; ^3^Department of Histology–Embryology, Medical School, Aristotle University of Thessaloniki, Thessaloniki, Central Macedonia, Greece; ^4^Department of Trauma Care and Patient Safety Education and Research, School of Nursing, University of Thessaly, Larissa, Thessaly, Greece; ^5^Department of Environmental Health, Harvard T.H. Chan School of Public Health, Boston, Massachusetts, USA

**Keywords:** Greece, health policies, legislation acts, rare diseases

## Abstract

**Background:**

Rare diseases are unique. Although they have low prevalence, they constitute a public health priority, affecting more than 350 million people worldwide. Patients are confronted with a complicated environment of sociomedical needs that require a strong public health response.

**Methods:**

European countries share allied multinational policies on rare diseases though inequities, delays in diagnosis, and differences between nations exist. Aiming to access the current Greek health policy, a thorough search on publicly published laws, governmental regulations, administrative information on orphan medical products, and associated actions was conducted. Regional health authorities' websites and relevant scientific webinars were extensively exploited and worked through.

**Results:**

Greece's national strategy for rare diseases includes creating a framework for screening, diagnosis, and treatment. The plan aligns with the EU recommendation to improve the visibility of rare diseases and ensure equal access to healthcare services. During the last 14 years, the Greek state has adopted the European definition and enacted targeted legislation so that, to date, 39 centers of expertise are certified and functioning, 3 disease-specific patient registries are actively operating, and 82 orphan drugs are reimbursed by the state.

**Conclusions:**

This analysis revealed gaps in core sectors: A national central patient registry is lacking, and interoperability between stakeholders can be further enriched and optimized, ensuring patient safety concerns. Health inequities for patients in remote areas should be addressed, and the orphan drug policy should be additionally improved. More research is needed to raise awareness and document patient-centered health policy tactics that drive wellness and treatment programs.

**Summary**

• Rare diseases (RDs) impact 260 million children and adolescents worldwide.
• Despite a common European Union (EU) legislative framework, national disparities and inequities remain.
• Several legal and social measures have been implemented over the past 15 years in Greece.
• Greece has made notable strides in rare disease care but still faces gaps in key policy areas.
• Further research is needed on young people's experiences within healthcare systems.

## 1. Introduction

According to the EU definition, RDs are considered those which affect 1 in 2000 people. RDs are characterized by low incidence and high heterogeneity, and most of them are genetic in nature [1]. Currently, 8000 RDs are recorded, and new RDs are identified and appended to scientific literature every day, with the number of new-onset RDs reaching 250–280 per year [2]. Although for some RDs the number of patients may be in the single digits or minimal, in aggregate, they affect a considerable number of patients, approximately 37 million people in the EU, 25 million in the United States, and 600,000 Greeks [3].

In many cases, RDs are multisystemic, debilitating, disabling, and potentially life-threatening. As 72% of RDs are of genetic origin and 75% occur in childhood and adolescence (0–19 years of age), their impact is felt way beyond the patient and strikes the family as well as the community. A total of 28% of RDs are nongenetic, mainly infectious diseases, but in most cases, a coexistence of genetic predisposition and environmental influence (exposure to infectious and toxic agents, dietary restrictions, drug interactions, etc.) is implicated, including rare cancers and health hazards [4]. Recent research highlights the complex epigenetic mechanisms that contribute to gene–environment interactions by presenting a striking RD: the recently identified acute multisystemic inflammatory syndrome in children (MIS-C) and adolescents associated with SARS-CoV-2 infection [5].

Due to their complicated etiopathology and clinical course, which is in most cases dynamic and unpredictable, RDs require a multifaceted environment of care and high-cost interventions including orphan drugs (ODs). The patient usually interacts with health professionals of different specialties (pediatricians, geneticists, laboratory biologists, developmental biologists, occupational therapists, speech and language therapists, general pathologists, etc.) and in separate health structures (from the community clinic and the family doctor to advance tertiary centers), resulting in uncertainty of care and unfulfilled patient and family care requirements [6]. Quantity and quality of unmet needs depend directly upon the characteristics and complications of the disease, the socioeconomic status of the family, the health services offered in the country, and the respective structure of the health system [7]. Diversity of healthcare systems makes a universal approach to RDs difficult, although the need for global policies and integrated action plans is well documented [8]. EU supports patients with RDs by providing its member countries with shared legislative frameworks, by funding research collaboration programs, and by having established *Orphanet* as the official web portal for RDs [9]. However, research reveals that European patients still face administrative barriers to accessing public health and social services and are often driven to catastrophic costs [10]. The National Strategy in several European countries seeks to involve all stakeholders, including patients, and to supervise the process of developing efficient and affordable systems for the treatment of RDs [11]. This task has been carried out in Greece since 2010.

In this paper, we aim to assess the current Greek health policy on RDs, identify gray areas, and finally evaluate the need for further research and associated actions. An exploratory online search of publicly published regulations, laws, government publications, associated actions, and other sources of pertinent OD information was conducted in order to obtain material within the article's focus. To do this, websites of regional health authorities (such as the Ministry of Health, the Institute of Child Health (ICH), the National Public Health Organization (NPHO), and formal patient organizations) were searched up, as well as an extensive manual search of relevant scientific webinars took place. This narrative review revealed no PubMed publications in Greece covering this area. In the following analysis, current policies for RDs in Greece are undertaken by cataloging, exploring, and commending on seven “core areas” related to RDs as defined by the EU [12]:

➢ Newborn screening (NBS) for RDs
➢ National Committee (NP) for RDs
➢ Centers of expertise (CE) for RDs
➢ RD patients' registries (central vs. disease-specific)
➢ Genetic and genomic services for RDs
➢ Access to ODs measured by the number of available reimbursed ODs
➢ Patient organizations and social policy

### 1.1. Greek Institute of Child Health (ICH) and National Neonatal Screening Program (NSP)

The labyrinthine pathway leading to the diagnosis of a RD has rightly been given the characterization as an “odyssey” (from the relevant Greek myth, referring to the “long and adventurous journey”). It is estimated that 40% of patients will require at least 5 years and undergo medical evaluations by five to eight different specialists, before a definite diagnosis is made. In the meantime, patients with RDs have received one or more misdiagnoses and corresponding treatments, including unnecessary surgical procedures and psychiatric medications [13]. As RDs are 72% genetic in origin and 75% involve children, it is of particular importance to screen for diseases that can be diagnosed at birth, the so-called neonatal screening in order to spare patients from the above long and unnecessary trouble.

In Greece, as in other Western countries, on the last day of a newborn's stay in hospital, a blood sample is taken for preventive screening for different diseases, whose early diagnosis will have multiple benefits to the future development of the child. The program started in 1974 as a pilot screening for phenylketonuria (PKU) and was gradually expanded with measurement of the activity of the enzyme of glucose 6-phosphate dehydrogenase (G6PD) and the control of congenital hypothyroidism (CH) and galactosemia (TGAL). According to the latest amendment of the Ministry of Health, these four RDs are currently being screened in the whole country [14]. Nevertheless, Greece is identified as one of the countries with the narrowest spectrum of diseases screened at present [15]. National Preventive Programs in other European countries vary with respect to the number of diseases that are currently checked (from 2 to 48), and effort is being made to harmonize practices. A NSP is not limited to a biochemical or molecular measurement but constitutes a comprehensive service grounding confirmation of the diagnosis and treatment and evaluating of the affected child's progress, according to the criteria of the World Health Organization (WHO) and the European Commission's Report [16]. Most European countries are adapting pilot applications for expanding their NSPs to a larger number of diseases (Table 1) [17].

According to the ICH in Greece, the importance of this screening is enormous, since “for the first two diseases,” early detection and appropriate treatment mean preventing cognitive impairment. The immediate diagnosis and treatment of the first two RDs are essential for the prevention of mental retardation that develops if these genetic problems remain untreated. For the third disease, it is necessary to undertake proper preventive and dietary measures to avoid fetal consequences. Up to date, more than 3,000,000 newborns have been screened, and more than 80,000 newborns are screened annually [14]. However, for the time being, the ICH does not perform extended neonatal screening examinations, such as those performed in Italy and Austria, which cover for 48 and 31 diseases, respectively. More importantly, the Greek NSP does not include RDs that are more prevalent in the Mediterranean basin such as thalassemia and cystic fibrosis [15].

The case of “free diagnosis of 500 genetic diseases with a drop of blood,” which was performed on 1000 infants in Greece in the “First Steps” study, involved the Papageorgiou Hospital in Thessaloniki, the Alexandra General Hospital in Athens, and the University Hospital of Larissa in August 2023. The processing of the samples was carried out at the Institute of Applied Biosciences of the National Center of Research and Technological Development (Thermi, Thessaloniki). The “First Steps” study utilized the whole human genome sequencing (WGS) method and analyzed about 400 genes associated with more than 500 genetic diseases. The study was part of a global initiative coordinated by Rady Children's Hospital (San Diego, United States) and was under the auspices of the Greek NPHO [18]. In addition, a very recent development is the approval of a 2-year pilot program to screen for 29 additional diseases (mainly metabolic) by the ICH (due to the donation of a needed laboratory equipment by Stavros Niarchos Foundation), which is expected to contribute to the screening biomethods in order to provide new results. Such an expansion will constitute an extremely positive development if applied to include 30 diseases on a permanent basis instead of the existing four [19]. According to Government Gazette 2433/*Β*/12-4-2023, Greece initiates a pilot application for the screening of 30 new diseases, including cystic fibrosis, as part of the expansion of the NSP [20].

### 1.2. National Committee for Rare and Complex Diseases (NRDC)

Following Directive 2011/24/*Ε*U, the Greek state issued and enacted Law 4213/2013 (Article 11), according to which Greece participates in the European Reference Networks (ERNs) developed at the European level with the support of the European Commission [21]. This law mandated the inclusion of RDs in cross-border care and was a fundamental step toward recognizing RDs, as it enabled patients with undiagnosed conditions and carriers of children with developmental problems or unclassified symptoms to legally seek help in tertiary/specialized European centers. According to Law 4213/2013 (Article 12), in the context of engaging in cooperation for diagnosis and treatment, there is a commitment to raising awareness and providing information to health professionals on issues related to RDs. By decision of the Minister of Health, Greece (a) establishes a permanent NRDC and defines its responsibilities and (b) recognizes *Orphanet* as the official database for RDs [22].

The Greek NRDC has been renewed three times since 2013, the last two under the umbrella of the Central Health Council (CHC). The second NRDC, whose mandate expired in January 2021, had to face a delay in its work due to the pandemic, and the third NRDC was established in November 2022 following pressure from patient organizations. According to Law 4461/2017 (Article 14), the CHC establishes the Greek “National Committee for Rare Diseases (NRDC),” which is permanent in nature and shall be composed of the following [23]:

➢ Three (3) health professionals and their alternates, proposed by the CHC, who must have a proven record of scientific work, experience in the management of RDs, experience in the management and operation of specialized health care units, experience in the management of specialized services, participation in relevant European programs and networks, proven experience in the evaluation of institutions, scientific activities, protocols, and active participation in relevant Greek, European, and international committees.
➢ The representative of Greece in the expert group on RDs of the European Commission.
➢ The Director in Public Health of the Ministry of Health, with his/her deputy.
➢ Two (2) representatives of patient organizations, with their deputies.
➢ Greece's representative to the European Commission's expert group on patient safety.
➢ Greece's representative to the European Commission's expert group on quality of health care for Europe and quality in health care, with his/her deputy.
➢ One (1) head of the quality improvement and quality control department of the public health services, with his/her deputy, appointed by the Minister of Health.
➢ One (1) representative of the National Organization of Medicines (E.O.F), with his/her deputy.
➢ One (1) representative of the National Organization for Healthcare Provision Services (EOPYY) with his/her deputy.

The Act provides for a 3-year office term for members and excludes members from membership of the committee who are employees or shareholders of pharmaceutical companies. The responsibilities of the NRDC are defined as follows:

➢ Formulation of a proposal for a National Action Plan for Rare Diseases with the aim of promoting the necessary legislation.
➢ Coordination and monitoring of the implementation of the proposed actions of the abovementioned national plan.
➢ Evaluation of the applications for recognition submitted by candidate CEs for Rare and Complex Diseases, in accordance with the criteria and conditions established by law.
➢ Five-year reassessment of recognized CEs.
➢ Declaration of activities for the last 3 years.

### 1.3. Centers of Excellence (CE) and Expertise for Rare and Complex Diseases

The existence of planning (primary diagnosis, navigation of the patient within the health system, follow-up, hospitalization, and involved therapists) and the supervision, monitoring, and coordination of the above processes constitute important milestones for effective management of each RD. CE and expertise for rare and complex diseases have acknowledged experience and provide high-quality services in the RD community [24]. According to Law 4461/2017 (Chapter B, Article 9), CEs are established as public health units that possess both proven expertise and scientific training for specific diseases and are supported by the appropriate laboratories to carry out the relevant biomedical tests. This ensures access to a multidisciplinary team interacting with global networks, implementing international treatment protocols and guidelines of renowned experts according to accredited scientific societies. That is, this law warrants networking with other Centers of Reference both nationally and internationally and wedges systematic record keeping of patients, incorporating their demographic and clinical information [23].

Hospitals that want to submit a nomination must file an application with specific criteria, measures, and documentation. The application follows an internal procedure described in detail in the law and is submitted by the applicant's medical director with the approval of the director of the sector as well as the chief medical service director, alongside the positive opinion of the scientific and administrative council of the hospital. A self-assessment team is recruited by the hospital's manager who also must exhibit a high level of commitment and assistance, expressed through active participation. In particular, the hospital manager takes over as a leader to participate in the decision-making process, while delegating responsibilities by setting up teams and supporting and supervising their actions. Finally, when specific criteria are scored, the leader submits the application file to the relevant health region which will forward it centrally to the Ministry of Health and finally to the national commission for RDs. The nomination form is checked for the level of completeness of the required information and is subsequently completed upon on-site examinations for the detailed evaluation of the quality of the data [25].

In Greece in 2019, 27 CE in RDs were recognized, and there are currently 39 CEs functioning, actually covering all human body systems (genetic disorders, congenital malformations, metabolic disorders, neurodevelopmental disorders, disorders of the nervous system, blood diseases, gastrointestinal diseases, diseases of the skin and subcutaneous tissue, diseases of the eye and its appendages, diseases of the respiratory system, diseases of the urogenital system, and metabolic diseases). Out of the above, 36 CEs are located in the capital city of Greece (Athens), at the National and Kapodistrian University of Athens, of which 6 concern children (Aghia Sophia Children's Hospital) [26].

### 1.4. RD Patients' Registries

The national registries (NRs) of RD patients encompass classification and coding of RDs, incorporating sociodemographic characteristics and clinical data of patients. NRs include patients with a view to protecting their privacy, in accordance with the provisions of the General Data Protection Regulation (EU Regulation No. 2016/679 of the European Parliament) [27]. NRs' construction and functionality depend upon cooperation with clinicians, academic organizations, patient groups, manufacturers, and regulators that enable the use of data to improve diagnosis and treatment. In this way, NRs streamline patient care pathways, reduce uncertainty, and support outcomes based on informed consent while ensuring patient confidentiality [28]. Most developed countries have NRs for RDs, but there is variation in their centralization, the range of diseases covered, and the amount of data collected. Four countries in Europe—Belgium, England, France, and Italy—have a single registry covering all RDs [29].

According to the website of the NPHO (EODY), “the registration of rare diseases in Greece is characterized as fragmentary.” The development of a national central RD registry is a priority in the Greek Ministry of Health, as it represents a constant key claim of associations and federations of patients and families. There are currently only three (3) disease-specific NRs for RDs: for patients (adults and children) with cystic fibrosis (launched in July 2020), for pediatric and adolescent patients with neoplasms (launched in February 2021), and for patients with spinal muscular atrophy (launched in April 2023). In order to gain the appropriate experience, the pilot registration of two more RDs is decided: Gaucher disease and Pompe disease [30].

### 1.5. Specialty in Medical Genetics

The new specialty of clinical and laboratory genetics was established in Greece in 2019 with the Government Gazette Issue B 2524/26.06.2019, necessary for the initial diagnosis of patients with RDs, the detection of the pedigree, and further guidance. A qualified physician can diagnose, provide prevention-genetic counseling, plan and administer proper treatment (gene therapy and other interventions), and predict illness course and survival expectancy. The evolution of medical genetics has followed the revelations of genomics such as deciphering the human genome and next-generation sequencing (NGS). NGS holds promise in becoming the primer diagnosing tool in the field of RDs [31]. The clinical geneticist evaluates the history; studies the patient's phenotype systematically, morphologically, and descriptively as well as the epigenetic factors; advises and recommends appropriate genetic testing; and responds to expectations of the family by assessing the risk of genetic recurrence in a subsequent pregnancy [32, 33]. A correlation is made between the detected DNA changes and the clinical phenotype, as there is also the phenomenon of genetic heterogeneity (different clinical symptoms of the same disease even within the same family). Incomplete penetration and diversity in gene expression in terms of symptom severity lead to the need to provide alternative methods of prevention during reproduction (proper prenatal/preimplantation screening, possibilities of targeted IVF, etc.) [33, 34].

Patients suffering from genetic diseases hospitalized in pediatric clinics are estimated at 40% of annual admissions. In large European genetic centers, intervention protocols are developed, where genetic counseling is combined with psychoemotional support of the family in making future reproductive decisions [35]. Genetic counseling takes its first steps in Greece, with the official establishment of the specialty, so that the state can gradually adjust a framework of institutionalized interventions embedding clinical, legal, and ethical viewpoints [33]. Clinical genomics and genetic counseling facilities currently gather in major tertiary centers in Greece, mainly located in the capital city of Athens.

### 1.6. ODs

Access for patients with RDs to innovative treatments is the big challenge for the Greek health system as budgets have been very tight due to the recent socioeconomic crisis (2008–2018), subsequent austerity measures, as well as the pandemic [36]. The single redundant insurance provider (EOPYY) is legally responsible to ensure patients' access to ODs and dispense them in its public pharmacies (EOPYY pharmacies). As the primary purchaser of healthcare services and medications, EOPYY establishes the terms necessary to acquire medical products and drugs at deeper discounts and generates enough space for economies of scale, guaranteeing unimpeded access to pharmaceutical products [37].

The payment of a certain amount to the public, and in particular to EOPYY and/or the Ministry of Health, by pharmaceutical companies that results from an annual overrun of the budget for EOPYY's pharmaceutical expenditure and the hospital's pharmaceutical expenditure is referred to as “claw-back” [38]. OD spending (excluding hospital drugs) was 3.6% in 2020, rising to 5.7% in 2023. For this reason, the health minister recently proposed a provision to treat ODs differently in terms of claw-back. The decoupling of ODs from hospital budgets, according to experts, will also spare medical professionals from having to constantly discuss with hospital managers whether to accept new patients. The Greek Health Ministry has recently proposed the design of parallel funding for ODs, in the form of an innovation fund, to better ensure patients' access to new treatments. Furthermore, pharmaceutical companies in Greece should be given incentives to develop ODs, which is becoming a mainstream European policy. Well-known European incentives for ODs, such as 10-year market exclusivity and patent deduction, are effective; nonetheless, there are variations in the methods for assessment, pricing, and compensation in different European countries. Notably in Greece, 74 ODs are distributed via the more costly emergency importation process through the Institute of Pharmaceutical Research and Technology (IFSET), whereas 82 ODs are currently reimbursed by EOPYY [39]. Government Gazette Issue 823/B 2.2.2024 regulates home delivery of specific ODs for patients suffering from the following RDs: cystic fibrosis, thalassemia, and ALS (amyotrophic lateral sclerosis) [40].

### 1.7. Patient Associations and Social Policy for Their Needs

The first patient associations in Greece began to emerge in the late 1980s. Given the extremely low prevalence of individual RDs, it was initially very difficult to gather the legally required minimum of 20 adult patients needed to form an official association with a constitution, board of directors, and registered members with defined roles and objectives. As a result, during the first phase (late 1980s to early 2000s), adult patients, parents, relatives, and close supporters aligned their efforts with the broader disability rights movement.

From the early 2000s onward—and in parallel with the rapid growth of RD associations and federations across Europe—Greek patients increasingly took leadership roles in advocating for access to medicines, treatments, and clinical protocols. During this second phase, a more structured and impactful patient movement emerged in Greece, primarily driven by parents of children and young adults affected by RDs.

By the mid-2010s, the RD movement had expanded significantly. A key milestone was reached in 2016 when the Hellenic Federation of Associations for Rare Diseases (H.F.A-R.D.) was recognized as a secondary social and trade union organization. Established in 2022, Rare Diseases Greece (RDG) serves as the national umbrella organization representing patients with RDs. It actively participates in key Greek committees and working groups—including the Committee for Rare Diseases, the Working Group on Patient Registries and Treatment Protocols, and the Pharmaceutical Expenditure Monitoring Committee of the Ministry of Health—as well as in international bodies such as EURORDIS and Rare Diseases International. Today, Greece hosts a diverse and dynamic network of nonprofit patient organizations (Table 2), which play a crucial role in policy advocacy, public awareness, and the delivery of essential support services.

These patient alliances are an integral part of the RD ecosystem in Greece. They not only offer direct support to patients and their families but also collaborate closely with healthcare professionals and policymakers to improve care pathways and treatment access. Members of these organizations are active participants in educational and scientific activities, including delivering lectures on epidemiology and gene identification, organizing training seminars for primary care physicians, and contributing to expert committees and policy forums [41].

A recent and noteworthy initiative is the development of the Hellenic RD map by the Hellenic Alliance for Rare Diseases, launched as part of the EMPOWER 2024 program. This innovative digital platform serves as a centralized resource offering reliable, Greek-language information on RDs and specialized healthcare services throughout the country. The map is aimed at addressing longstanding information gaps, facilitating equitable access to care, and supporting healthcare professionals with accurate and up-to-date data, ultimately helping to alleviate the geographic and informational isolation experienced by many patients with RDs [42].

The needs of young RD patients intersect multiple public sectors. The Ministry of Health is responsible for timely diagnosis, therapeutic interventions, and long-term care. The Ministry of Education addresses issues such as the assignment of special education teachers, provision of assistive technologies, and special admission procedures for university entrance. The Ministry of Labor deals with employment-related matters, including parental leave, disability certifications, and financial subsidies.

Although 2016–2017 marked a period of increased recognition and awareness of RDs in Greece, much of this momentum was lost during the COVID-19 pandemic. Revitalizing this awareness—and fully leveraging new initiatives such as the RD map—remains a pressing challenge for the Greek National Health System (NHS) today.

“Social policy” refers to a set of government actions aimed at promoting societal well-being, historically linked to health and welfare systems, most notably the Beveridge model. Originating from the 1942 report *Social Insurance and Allied Services* by economist William Beveridge, this model laid the foundation for the postwar British welfare state, including National Insurance and the National Health Service. Under this system, healthcare is primarily state-funded through taxation, with publicly owned and staffed facilities, although private providers may also be reimbursed by the public sector [43]. By eliminating commercial market competition, the Beveridge model helps maintain cost control and ensures free access to services at the point of delivery. While the model has distinct features, its principles have been adopted or adapted in countries such as Norway, New Zealand, Denmark, Italy, and Spain. Greece, though characterized by a mixed health system, integrates several Beveridge-inspired elements within its public healthcare network [44].

In the context of chronic and RDs, social policy addresses key issues such as the organization of care, funding mechanisms, institutional structures, and bureaucratic processes involved in treatment delivery. However, the perspective of the care recipients, particularly children, adolescents, and the elderly, is often overlooked. Since Law 1416/84, which initiated elderly day care services [45], and Law 4483/2017 [46] regulating home care services, various ministerial decisions have established institutional frameworks across public and private sectors. Yet, these mostly target psychiatric and geriatric care. Overall, Greek social policy remains weakly institutionalized, lacking universality and equity, with caregiving responsibilities largely falling to families—particularly women [47]. This narrative review reveals a significant lack of research on the psychosocial needs of RD patients in Greece. Specifically, there is an absence of literature documenting the lived experiences of children and adolescents within the Greek NHS.

## 2. Discussion

During the past 15 years, the NHS in Greece suffers from multiple crises including reduced public health spending, immigration of medical professionals, salary decreases, lower purchases of medical supplies, reforms in the social insurance and pharmaceutical industries, and a significant rise in unmet health needs that have been documented [48]. Though a more comprehensive analysis goes beyond the scope of this paper, it is worth pinpointing that the NHS has been profoundly impacted by a series of successive crises, notably the prolonged economic recession, the implementation of austerity measures, and the COVID-19 pandemic. The financial crisis and ensuing bailout agreements necessitated substantial reductions in public health expenditure, which in turn curtailed access to essential medical services [49]. Hospitals faced persistent shortages of medical supplies and personnel, while primary healthcare services were increasingly strained under rising demands. Patients with chronic and RDs were particularly vulnerable, as access to specialized treatments diminished and preventive care initiatives were deprioritized [50]. The COVID-19 pandemic further intensified these structural deficiencies by diverting resources toward emergency response efforts, thereby exacerbating delays and inadequacies in care for non–COVID-19 patients [51].

Concomitantly, the NHS fails to holistically address chronic diseases, as the models of integrated care are still developing, despite relevant political intentions and Europeanization. The hospital-centric nature of the system with a lack of sufficient focus on primary health care prevents effective development of integrated and coordinated health care service delivery [52]. Moreover, the specific geographical morphology of Greece with a mountainous mainland, several remote villages, and many small, scattered islands also exacerbates the already existing difficulties regarding accessibility to health care, especially for vulnerable populations who reside in remote areas. In addition, while public services exist in long-term nursing care institutions, the official home care arrangements are limited and have mainly been associated with geriatric patients. Likewise, targeting research on chronic diseases focuses on more common health problems (diabetes, asthma, and heart diseases) and elderly patients (e.g., those with dementia), neglecting patients with RDs, especially children, adolescents, and young people [53]. In this paper, we explored and summarized the Greek health-related policy decisions in the field of RDs in seven fundamental areas (NBS, national committee, CE, RD patients' registries, genetic and genomic services, patients' associations, and access to ODs).

It is estimated that approximately 600,000 people in Greece suffer from RDs, of whom 400,000 are children and young people. Greece has recognized the European RD definition since 2010 and has directly developed the first National Action Plan within the framework of the CHC inside the Ministry of Health. The first National Committee on Rare Diseases incorporated the diagnosis and treatment of RDs in Law 4213/13 [22] on cross-border care and acknowledged the *Orphanet* portal, resulting in recognition of RDs. Between 2017 and 2025, Greece established 39 CEs, passed legislation concerning patients' medical, educational, and psychosocial needs, and currently distributes 156 ODs. Scientific literature recognizes worldwide disparities in diagnosing, monitoring, treating, and emotionally supporting rare patients, since sociomedical services are geographically dispersed and CEs are mainly placed in tertiary hospitals in big cities [54]. This is also the case in Greece hosting from the 39 available CEs: 36 in Athens, 1 in Thessaloniki, 1 in Larisa, and 1 in Patra, according to the latest data from the Greek Ministry of Health [55]. This narrative review identified deficiencies in four essential areas: Health inequalities affecting patients in remote regions should be addressed, there is an absence of a national central patient registry, the interoperability among services can be enhanced and optimized, and there is a need for further encouragement of OD policies.

Although Greece has engaged in European collaborations and developed a National Action Plan for Rare Diseases, its implementation remains inconsistent. Considering evolving EU policies, advances in genomic medicine, and emerging digital health tools, Greece stands at a critical juncture to redefine its RD policy framework and answer key policy challenges:

a. Fragmented care pathways

Patients often navigate a disjointed system without coordinated referral protocols, case management, or integrated service delivery. Multidisciplinary care remains limited to a few specialized centers.

b. Delayed diagnosis and lack of expertise

On average, RD patients in Greece wait over 5 years for an accurate diagnosis, reflecting a broader scarcity of trained specialists and limited diagnostic technologies.

c. Data deficiencies and registry gaps

Greece lacks a centralized, interoperable national RD registry, undermining research, epidemiological tracking, and healthcare planning.

d. Restricted access to ODs and therapies

Orphan medicinal products face reimbursement delays and regulatory barriers. Fiscal austerity measures have also contributed to inconsistent availability.

The ICH was coordinating the Greek national project for *Orphanet* from 2008 to 2014, a period during which data from several RDs were translated into Greek. The project was discontinued in 2014, due to a lack of sufficient funding, resulting in inadequate exploitation of the portal's potential. Several rare patients had to prove their disease to the services involved, whereas key information should be easily manageable through digital functional programs. Uncharted patients faced discontinuity in the prescription of vital treatments and their reimbursement by the state.

Health data set structuring, collection, and recording regarding patients with RDs constitute a global challenge, since this kind of data is, by definition, voluminous and complex [56]. Greek patients with RDs are constantly fighting to achieve consensus for homogeneous data collection at the point of care and validation of interoperability between services and demand the formation of a central national registry. Nevertheless, planned construction of additional disease-specific NRs (three are currently in function) has started and is expected to create an open channel of communication with the CEs so that diseases can be codified and manipulated in practice. Any expenditure or benefit for the individual's Social Security number will be recorded and administratively distributed. Visibility from the health system will make it easier to further manage RDs, so that epidemiological and clinical data will be interdisciplinary and will be classified among therapists and service providers. This procedure will enable the single insurance provider EOPYY to automatically recognize patients and pay the statutory benefits for each of them accordingly [57]. A recent positive development enhancing the above target is the fact that with Government Gazette Issue 248/B 17.1.2024, the Greek list of RDs has been initiated and is scheduled to fully interoperate with ICD-10 (the standardized system used by healthcare professionals to define diagnosis and to prescribe medical products) as well as with other biomedical software by the end of 2024 [58]. In conclusion, our review culminated in the formulation of a series of policy recommendations.

## 3. Strategic Policy Proposals

Addressing the needs of individuals living with RDs constitutes both a moral imperative and a strategic health opportunity. A modern, patient-centered RD policy in Greece should promote equity, foster innovation, and ensure sustainability. By aligning with European frameworks and leveraging advances in genomics and digital health, Greece can provide timely, coordinated, and effective care to one of its most vulnerable populations.

### 3.1. Develop a National Strategy for RDs (2025–2030)

A revised, comprehensive national strategy should include measurable objectives, clear governance structures, and budgetary commitments. The strategy should align with the European Commission's current RD roadmap.

### 3.2. Integrate Registries and Digital Infrastructure

Creating a unified national RD registry linked with the electronic health record (EHR) system is of paramount importance, along with ensuring compliance with EU data protection and interoperability standards (e.g., HL7 FHIR).

### 3.3. Expand Genetic and Genomic Diagnostic Services

Invest in national genomics programs and NGS platforms. Establish regional hubs to reduce geographic disparities in diagnostic access.

### 3.4. Recognize and Fund CE

Formalize resource-selected hospitals and research institutions as National Centers of Expertise, with criteria harmonized with ERN standards.

### 3.5. Institutionalize Patient Engagement

Codify the role of patient organizations in decision-making processes including health technology assessments (HTAs), clinical guideline development, and service design.

### 3.6. Secure Dedicated and Sustainable Funding

Establish protected funding lines for RD care and OD reimbursement. Encourage innovative funding models such as outcome-based pricing and managed entry agreements.

### 3.7. Enhance Workforce Training and Public Awareness

Introduce mandatory modules on RDs in undergraduate medical curricula. Provide continuous professional development (CPD) for physicians, pharmacists, and allied health professionals.

## 4. Study Limitations

Our study offers a valuable overview of key policy achievements and recognizes deficiencies within the Greek health policy landscape regarding patients suffering from RDs; however, several limitations warrant consideration. The analysis primarily emphasizes the identification and description of policy gaps, without delving deeply into their underlying causes or advancing detailed, evidence-based policy interventions. As such, the paper should be regarded as a preliminary narrative review making key point suggestions, rather than a comprehensive policy framework assessment. Moreover, while the national scope enhances contextual relevance for Greece, it may constrain the generalizability of the findings within a broader European context. Future research could build upon this foundation by integrating cross-national comparative analyses, examining systemic determinants, and formulating actionable policy recommendations aimed at improving health policy effectiveness.

## 5. Conclusions

Greece, a developed European country, follows the European Directives in the field of health and in the challenging case of RDs. RDs are characterized by specificities in diagnosis, multisystemic treatments and require advanced clinical and laboratory surveillance and monitoring. Access to affordable and comprehensive medical and psychosocial care for patients with RDs emerges as a public healthcare priority. Patients with these conditions are, by definition, an example of e-patients that are daily dependent on their sensitive data to be securely available across multiple health care providers. This narrative analysis is unique—to our knowledge—to present good practices developed by the Greek state in the above areas. Our review emphasizes the latest legislative achievements, setting priorities across a patient-centered approach and highlighting the need for further research, especially through the living experiences of patients and their caregivers.

## Figures and Tables

**Table 1:** Preventive neonatal screening in selected European countries (adapted from [17]).

**European countries**	**Diseases tested in national panel**	**Pilot or scheduled implementation**
Italy	48	60
Austria	31	31
Portugal	30	31
Poland	29	33
Hungary	27	28
Sweden	27	27
The Netherlands	27	31
Norway	26	26
Slovakia	26	26
Denmark	25	25
Finland	23	24
Estonia	22	23
Germany	21	21
Ukraine	21	21
Czech Republic	20	21
Slovenia	19	21
France	13	15
Belgium	12	23
Lithuania	12	12
Switzerland	10	11
Croatia	10	10
UK	9	11
Spain	8	41
Ireland	8	10
Latvia	8	8
Luxembourg	6	6
Malta	5	5
Greece	4	30
Bulgaria	4	4
Romania	3	4
Cyprus	2	10

**Table 2:** Key patient organizations in Greece that focus on rare diseases.

**Organization**	**Focus area**	**Headquarters**	**Website**
Rare Diseases Greece (RDG)	Umbrella organization for rare disease patient groups	Athens	http://rarediseasesgreece.com
Greek Patients Association (GPA)	National coalition representing patients and carers across various diseases	Athens	greekpatient.gr
Panhellenic Association of Patients with Lysosomal Disorders	Lysosomal storage diseases (e.g., Gaucher and Fabry)	Gerakas, Athens	greeklysosomal.gr
Hellenic Cystic Fibrosis Association (HCFA)	Cystic fibrosis	Athens	cysticfibrosis.gr
Hellenic Friedreich's Ataxia Association (HEFAA)	Friedreich's ataxia	Thessaloniki	http://hefaa.org
Karkinaki	Childhood and adolescent cancer awareness and support	Athens	karkinaki.gr
Angelman Syndrome Greece	Support for families affected by Angelman syndrome	Galatsi, Athens	angelman.gr
VHL Family Alliance Greece	Von Hippel–Lindau disease	Athens	http://vhlgr.org
Hellenic Pulmonary Hypertension Association	Pulmonary hypertension	Athens	http://hellenicph.org

## Data Availability

Data is available from the corresponding author on reasonable request.
